# Does It Pay to Issue Green? An Institutional Comparison of Mainland China and Hong Kong’s Stock Markets Toward Green Bonds

**DOI:** 10.3389/fpsyg.2022.833847

**Published:** 2022-04-14

**Authors:** Xingxing Chen, Olaf Weber, Vasundhara Saravade

**Affiliations:** ^1^School of Economics and Management, Wuyi University, Jiangmen, China; ^2^School of Environment, Enterprise, and Development, University of Waterloo, Waterloo, ON, Canada

**Keywords:** stock market, investor reaction, green bond, event study, comparison

## Abstract

The stock market is an indicator of investor sentiment when it comes to new information or innovative firm-level products. Green bonds are both innovative and unique in terms of their higher information disclosures and understanding the impact of sustainable finance on investor outlook for a company’s stock. Using the comparative case of Mainland China and Hong Kong’s stock market, we examine whether green bond announcements from 2016 to 2019 can create significant investor reactions. By employing the event study methodology, we confirm that both markets react in a positive way toward green bond announcements. This reinforces the reputational and financial benefits of green bonds. We find that issuers that are non-banks, environmentally friendly firms as well as those issuing non-general bonds, create a more positive reaction, whereas ownership aspects do not matter as much for investors. However, even among those issuers listed in both markets, certain institutional dynamics like strategic framing and source credibility tend to reinforce a firm’s institutional legitimacy and are seen as being more prominent for investor reaction. The policy implications of our study show that the stock market reaction among two connected economies, where previously varying institutional contexts have resulted in regional differences, are now equally supportive of sustainable financial markets like the green bond. As seen with the positive stock market sentiment, governments and listed issuers can now better align their policies and internal strategies, allowing the low-carbon transition to be a financially attractive opportunity for all investors.

## Introduction

The green bond market in China has been consistently outperforming other developed and emerging economies since the last few years. Following its inception in 2016, China’s green bond market has been backed by a range of government policies and institutional incentives since the very beginning. For instance, not only does the market ecosystem exist in terms of domestic verification and regulations for green bonds, but other transition-linked aspects of the economy like green credit and carbon pricing are also present in China (Cui et al., 2018). Given this top-down-driven push toward a low-carbon climate resilient (LCR) economy, China’s green bond market has boomed based on the right financial and institutional incentives (Huang and Yue, 2020).

However, in terms of capturing the real impact of green bonds in China, there is a need to understand whether this positive development translates to other financial markets, like the stock market (Baulkaran, 2019). Furthermore, since abnormal returns on stocks often reflect investors’ current attitudes (Kurov, 2010; Akhtar et al., 2012; M’ng et al., 2019), there is a need to distinguish stock market reactions of different regions or countries because it can help identify investor sentiments and regulator policy implications for green financial products. For instance, the question remains whether the Chinese green bonds market is driven exclusively by government regulations or because of the business case for addressing climate risk and other pressing environmental problems. To understand the stock market impact of green bonds, our paper compares the Mainland China stock market with that of Hong Kong and tries to pinpoint whether an announcement related to green bond issuances might have a varying impact based on the institutional differences or similarities of the two markets.

Although the stock market is focused on the short term, it has previously been related to the long-term bond market in uncertain and contradictory terms. Some studies are in favor of the hypothesis that bond issuance announcements have a positive and signaling effect for shareholders (Ross, 1977; Chin and Abdullah, 2013; Tang and Zhang, 2020), whereas others suggest that it can either increase the financial risk or cannot be correlated with it at all (Masulis, 1983; Myers and Majluf, 1984; Miller and Rock, 1985). However, a more recent study examining three emerging ASEAN countries indicated that there is a significant effect of bond announcements on share price returns of the issuers and that issuers can use this as a signaling opportunity to change investor perception (M’ng et al., 2019). This is important because if a bond market announcement correlates with a stock market reaction, there are clearly financial and reputational repercussions for the bond issuer. Hence, examining the green bond market provides a unique case study to document how these reactions might occur if the stock market prefers a sustainable finance tool that had a climate change strategy associated with it.

As suggested by the efficient market hypothesis, financial markets react to all available information by incorporating it efficiently into the stock price without any delay. However, many studies (Akhtar et al., 2012; M’ng et al., 2019) show that investors can be irrational when it comes to sentiment, and this further drives stock price fluctuations around a specific event (Paskaleva and Stoykova, 2021), based on its overall perception as being positive or negative (Akhtar et al., 2012), which is further shaped by the narrative surrounding it as well (Kurov, 2010). For instance, studies analyzing stock prices around corporate social responsibility (CSR) announcements show that there can be a positive or negative investor reaction (Yu, 2012; Cordeiro and Tewari, 2015; Du et al., 2017; OuYang et al., 2017) based on the narrative at the time. For example, on the day of the environmental disaster of the Deepwater Horizon oil spill, the stock for British Petroleum rose from US$59.48 to US$60.48; however, after a stream of negative publicity coverage and the threat of changing liability laws and environmental regulations over the next few months, the stock price dropped at a low of US$ 27.02, which further impacted its share value by almost 38 to 41% over the event period (Boudreaux et al., 2013).

Given the changing narrative around what is acceptable and what is not (with climate impacts becoming more material to investors and governments), it can ultimately enhance or diminish the share value. Hence, our paper addresses market perceptions around sustainable finance tools like green bonds and whether they can create any significant reactions in two different sociopolitical systems’ stock markets. This is useful because green bonds are seen as a long-standing success story within the sustainable finance markets. Hence, we seek to understand how specific institutional policies are driving the market (like in the case of Mainland China and Hong Kong) and whether green bonds provide reliable climate-related financial information for those based in a short-term, sentiment-driven stock market.

Some of the most commonly studied variables in previous bond announcement stock reaction event studies have been the influences of firm characteristics such as company size, asset tangibility, profitability, growth opportunities, business sector or industry type, and ownership (Chin and Abdullah, 2013). In this study, we contribute to this literature by examining the influence of specific key characteristics like issuer type (bank vs. non-bank), ownership (private vs. government-owned), industry type (environmentally friendly vs. others), and bond type (general corporate, financial corporate, and non-general bonds) in the context of China and Hong Kong’s stock market reactions to corporate green bond announcements. The results suggest that stock investors responded positively to companies issuing green bonds for both Mainland China and Hong Kong stock markets. Certain issuers like non-banks and environmentally friendly firms as well as certain bond types like non-general bonds achieve a significant positive reaction among stock market investors. In contrast, differences in ownership characteristics are not significant to the market reaction. The policy implication is that policymakers can engage more actively in the market by creating green incentives and regulations that seek to increase investor confidence and issuer participation. A second implication is that it provides a tangible financial benefit for listed issuers that are looking to transition toward a green business model, especially when it fits in line with the institutional dynamics of the low-carbon economy.

The paper structure is as follows: firstly, we highlight the policy and historical background of the two stock markets and their green bond markets and outline our research questions. We then review the literature focusing on green bond markets and stock market investor sentiment. In the sample and methods section, we outline our event study sample and explain how we used it to address our hypothesis. Our results section highlights descriptive quantitative data, the results of the statistical tests, and tests for robustness. We then present a discussion section that highlights how this study contributes to the literature on stock market reactions of investors around green bond announcements as well as how they compare in the two different institutional contexts. Finally, we present our conclusion and policy recommendations.

## Background

**Figure 1:**
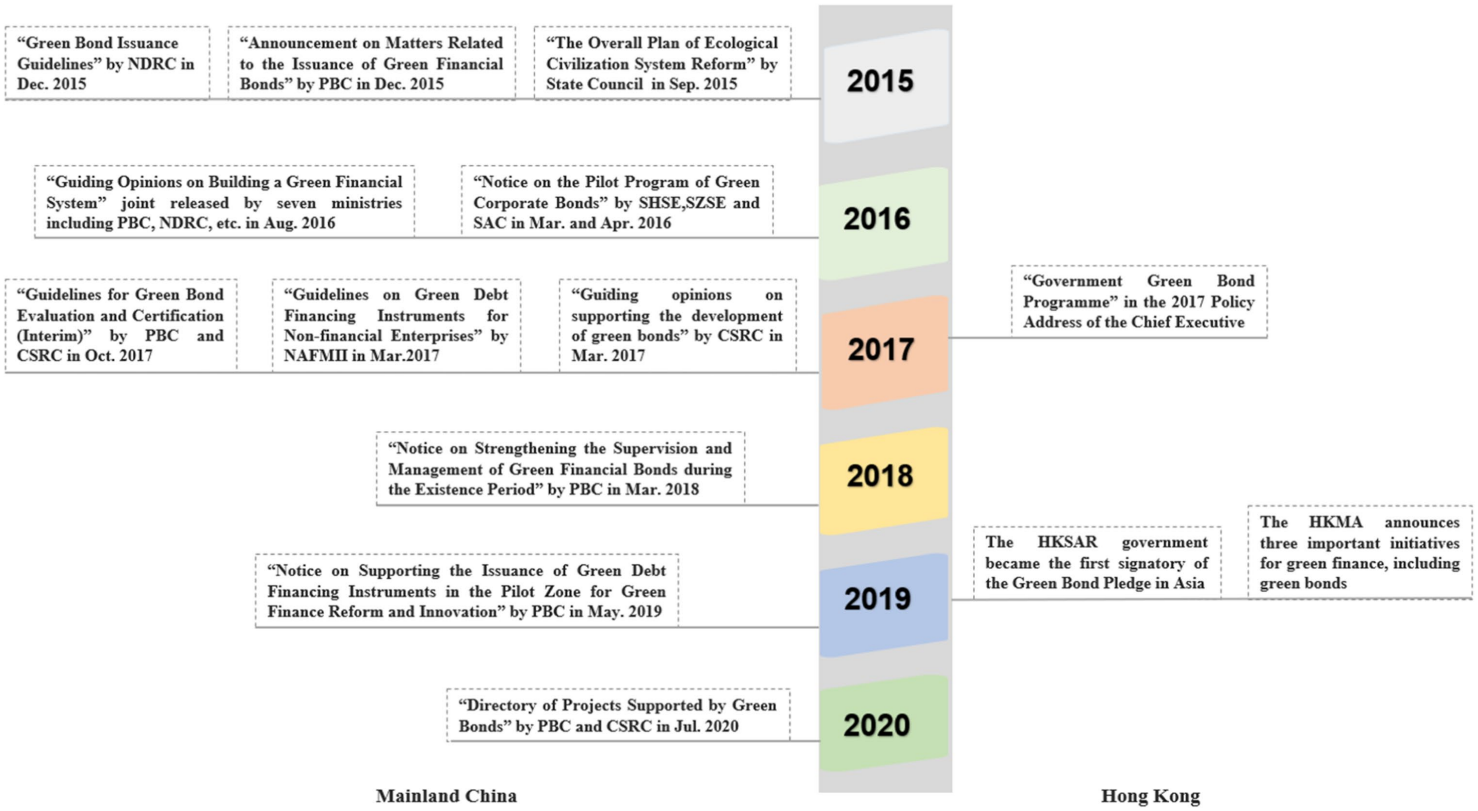
The overview of green bond regulations in Mainland China and Hong Kong from 2015 to 2020.

The rapid development of China’s green bond market has benefited from strong policy support. As shown in Figure 1, the Chinese government and regulators are actively involved in supporting green bonds’ institutionalization since 2015. In September 2015, the State Council of China issued the “Overall Plan for the Reform of Ecological Civilization System,” which proposed to encourage banks and enterprises to issue green bonds (State Council of China, 2015). Whereafter, bond regulatory authorities released guidelines for the issuance of different types of green bonds, including the People’s Bank of China (PBC), the National Development and Reform Commission (NDRC), the Shanghai Stock Exchange (SHSE), the Shenzhen Stock Exchange (SZSE), the Securities Association of China (SAC), the China Securities Regulatory Commission (CSRC), and the National Association of Financial Market Institutional Investors (NAFMII). Moreover, there are corresponding policies on the evaluation and certification, information disclosure, and green bonds’ duration supervision.

**Figure 2:**
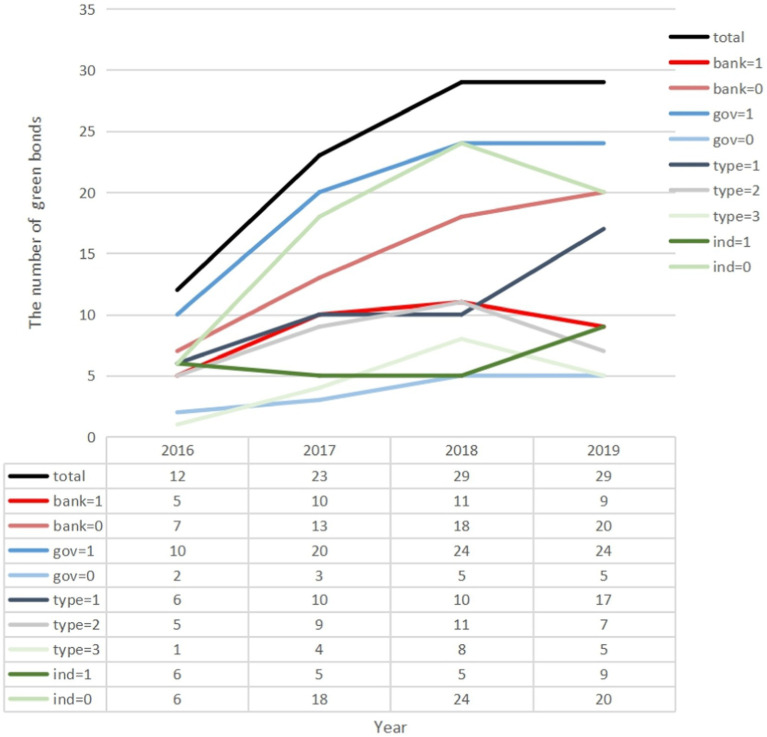
Sample composition of firm types, industry types, and bond types.

According to the policy documents entitled “Green bond issuance guidelines” released by the National Development and Reform Commission (NDRC), if the bond issuers plan to invest the raised capitals into environmental-friendly projects, then they can apply to issue green bonds. Those projects include technological transformation of energy conservation and emission reduction, green urbanization, clean and efficient utilization of energy, development and utilization of new energy, development of circular economy, water resources conservation and development and utilization of unconventional water resources, pollution prevention and control, ecological agroforestry, low-carbon economic development, energy conservation and environmental protection, ecological civilization pilot demonstrations, and low-carbon pilot demonstrations. This notion of green bonds is the basis on which China’s bond regulator approves whether companies can label their proposed bonds with a green label. With this standard definition in place, China’s green bond market has grown rapidly.

Green bonds in Hong Kong are also becoming an important investment vehicle to direct capital into green sectors. For instance, the Hong Kong Special Administrative Region (HKSAR) government issued its inaugural green bond in 2019, and regulators such as the Hong Kong Market Authority (HKMA) and the Securities and Futures Commission (SFC) are getting involved more actively. HKMA is responsible for overseeing the Hong Kong Stock Exchange (HKSE), which actively tracks the green bond growth and does capacity building around green finance implementation (Lau et al., 2020). SFC has implemented enhanced disclosure requirements on environmental, social, and governance (ESG) issues and provides an opportunity for listed companies issuing green bonds and fulfilling their disclosure requirements (Lau et al., 2020). More recently, in 2019, the HKSAR government became the first signatory of the Green Bond Pledge from Asia, and HKMA also joined the Central Banks and Supervisors Network for Greening the Financial System (NGFS).

Figure 1 demonstrates that green bond-related policies in Mainland China are more direct and frequent than in Hong Kong, which has created stronger institutional pressure on bond issuers. Regarding the influence of government policy on shaping financial markets, unlike other countries, Mainland China’s bond trading market was not just created by market forces, but rather by its government (Wang et al., 2020). Many financial institutions involved in the bond market are also state-controlled. Hence, this raises the question of the sociopolitical factors driving the development of this market—let alone the green bond market (Huang and Yue, 2020; Wang et al., 2020). Huang and Yue (2020) show that green bond issuances from state-controlled banks and enterprises are seen as good behavior to gain political credit and further stimulate the growth of China’s green financial system. Hence, the green bond market in Mainland China is intrinsically linked to the political environment in terms of policy, legal, and social aspects (Huang and Yue, 2020).

On the other hand, Hong Kong’s green bond market links to its political environment have not been examined much in the literature. However, in general, the Hong Kong market is seen as being more mature and in tune with international markets (Chiou and Lee, 2013; Sheng, 2018). However, since the mid-1980s, the financial markets for this region have been “increasingly integrated due to unilateral direct investments in China from private sectors in Hong Kong” (Chiou and Lee, 2013, p. 342). The greater integration is also witnessed with the two economies signing the Closer Economic Partnership Agreement (CEPA) policy in 2003. In addition, in 2014 the Stock Connect Scheme was launched. This launch further integrated the stock markets of Hong Kong and Mainland China allowing international investors direct access to Mainland China’s two stock markets based in Shanghai and Shenzhen, as well as to Chinese assets through the Hong Kong Stock Exchange (Cheng et al., 2019).

Regarding the maturity of the economy and its stock market, the volatilities and mean–variance efficiency of stock returns in Hong Kong are less risky than those in Mainland China, indicating that Hong Kong is a more mature economy overall (Chiou and Lee, 2013). The institutional influence is also reinforced by “Hong Kong [being] one of the world’s ten largest banking centers, the fifth largest forex markets, and the third largest stock market, [as well as having] more foreign banks in Hong Kong than in Singapore and Japan combined” (Sheng, 2018, p. 173). Being such an international finance center indicates the strong institutional policy factors that are now shaping Hong Kong’s stock market reactions and how they might differ from those in China.

In the two different markets, the development of green bonds shows different characteristics. In Hong Kong, the green bond market began in 2016 and has had a cumulative issuance size of US$2.6 billion by 2019. Mainland China, on the other hand, has been leading the total issuance size with a collective amount of US$21.8 billion by 2019. Both markets have a high rate of third-party verification of their green bonds, but Hong Kong (85%) does slightly better than Mainland China (74%). The Mainland China market has a high number of corporations (both financial and non-financial) that prioritize investment into low-carbon transport and energy sectors. In Hong Kong, the main sectoral driver has been the low-carbon building sector, and higher issuer participation has been from financial institutions like banks (Climate Bonds Initiative, 2019). The two markets differ in terms of the total issuance size, priority sector for the use-of-proceeds, issuer types, and number of third-party reviews of green bonds.

Apart from institutional pressure through government and regulators, other influences driving the rapid development of green bonds in both markets may be discovered from the comparison between mainland China and Hong Kong. Given the different institutional settings and the more recent efforts to integrate into a cohesive regional economy, it is important to understand what is driving stock market investors’ reactions when it comes to green bonds. To identify these different institutional drivers, our paper asks the following research questions:

- What is the overall investor reaction to green bond announcements in Mainland China and Hong Kong’s stock markets?
- How do issuer- and bond-level characteristics influence the stock investor reactions in both markets?
- How does the institutional standing of firms listed in both markets influence the stock investor reaction?

## Literature Review and Hypotheses

Recent literature on the topic of green bonds is only starting to emerge. To answer our research questions, we use literature looking at the green bond market, the connection between stock and bond markets, investor sentiment research, and the overall institutional policy contexts of Mainland China and Hong Kong. Our hypotheses are outlined below and are based on the above highlighted literature.

*H1*: Green bond announcements from firms lead to a positive reaction in both stock markets.

Not only has the green bond market literature grown in the last 5 years, but the scope of the research has broadened as well. Some studies have focused on the benefits such as positive financial and environmental performance of corporate green bonds (Tang and Zhang, 2020; Flammer, 2021). Others have identified potential drawbacks such as pricing differentials (Hachenberg and Schiereck, 2018; Zerbib, 2019; Guo and Zhou, 2021) or lower returns between green and conventional bonds (Bachelet et al., 2019; Larcker and Watts, 2020). Other studies have addressed green bond markets as an opportunity for both investors engaging with green finance (Chiesa and Barua, 2019; Saravade and Weber, 2020) and for countries that will face significant climate risks in the future (Broadstock and Cheng, 2019; Chiesa and Barua, 2019; Fatica and Panzica, 2021). To support the transition to a low-carbon economy, major institutional actors such as investors and governments have started to issue green bond policies and green fiscal measures to reduce climate risk exposure (Monasterolo and Raberto, 2018; Hunt and Weber, 2019; Saravade and Weber, 2020; Fatica et al., 2021).

Although only a few studies address the stock market reaction to green bond market announcements, there is a need to contextualize such findings based on different market types. For example, Lebelle et al. (2020) discuss how developed stock markets react more negatively to green bond issuances than emerging markets. However, they do not explain the differences in the reaction of these two market types. Wang et al. (2020) find that there is a greater pricing premium for Chinese green bonds relative to conventional bonds, especially in the global market setting. The finding shows that Chinese investors see the green bond market more positively, and green bond announcements are perceived as a positive signal in both stock markets.

*H2*: Reactions in the two markets will be influenced by issuer characteristics (type of issuer, ownership, and industry type).

Although both markets might react positively to the issuance of a green bonds, the reactions might be influenced by issuer- or firm-level characteristics. Recent research from Wang et al. (2020) connects China’s green bonds with stock market reactions, yet the contextual nature of why and how these reactions are occurring is currently missing. Furthermore, we still need to understand whether the reaction is unique to the Mainland or applies in other regional Chinese stock markets.

Wang et al. (2020) find that new green bond issuance announcements in China create positive stock reactions based on stakeholder value maximization and corporate engagement to increase firm value in the long run. However, Baulkaran (2019) suggests that global stock market reactions to green bond issuance announcements can differ based on firm-level characteristics. For example, aspects such as a higher coupon rate of the green bond or operating cash flow seem to have a negative reaction. Other aspects such as firm growth, firm size, and Tobin’s Q seem to have a positive reaction (Baulkaran, 2019) among global investors. This result indicates that firm and bond characteristics will likely matter when it comes to investor reactions to green bond announcements in both markets. However, given that the Hong Kong market is more international and might have non-Chinese investors, their reactions might differ from those in Mainland China.

Cordeiro and Tewari (2015) also find in the context of corporate social responsibility (CSR) performance (such as the issuance of a green bond) and investor reaction, that large and visible firms benefit more in terms of a stock price increase. This is supported by OuYang et al. (2017) who correlate corporate media reputation in China with stock market investor reaction in the time of a corporate crisis. They highlight how signaling appears in the media as a key intermediary in highlighting firm quality and reputation to investors, especially in times of corporate crisis or significant change. Kurov (2010) adds to this by showing how “attention grabbing events effect the information of investor’s mental reference levels assigned to stocks… and as such, the tone and volume of media reporting on the economy affects the consumer sentiment more than economic fundamentals would suggest” (p.144). Hence, firm- or issuer-level characteristics surrounding the issuer’s reputation and signaling strength might also play a role in the two markets’ reaction. With Hong Kong being a major financial center of the world, the level of media exposure of green bonds may be higher than that in Mainland China. Hence, we anticipate a different investor reaction to news of green bond issuances in Hong Kong compared to Mainland China’s stock markets.

*H3*: Reactions in the two markets are influenced by bond characteristics (general corporate, financial, and non-general bonds).

Wang et al. (2020) show that bond characteristics like debt credit ratings, issue period, and issue size are three different factors that directly affect the risk premium of green bond issuances in China. However, Zhou and Cui (2019) also reveal how corporate performance and socially responsible investing (SRI) related to environmental issues affect the expansion of the Chinese green bond market positively. To support this, Flammer (2021) finds that verification or governance mechanisms, such as third-party reviews, are a main characteristic for their global popularity among investors. Other specific bond characteristics like improvement in credit quality, lower cost of capital for bond issuers, and state support are also main drivers for green bond growth (Agliardi and Agliardi, 2019).

In China’s specific context, having a non-general bond category (or known as a “super & short-term commercial paper”) is a unique feature of the bond market. Such bonds usually have a term of 270 days or less and can be issued by a non-financial corporate with a high credit rating (Asian Development Bank, 2011). Not only are they more innovative in nature but they are primarily used to provide the issuer more liquidity than a short-term bond (Asian Development Bank, 2011). Given the backing by China’s National Association of Financial Market Institutional Investors (NAFMII), innovative characteristics of the non-general bond category are a way to help firms improve their liquidity and optimize product design. Hence, this implies that bond-level characteristics such as type of bond, issue period, and third-party verification (specifically for green bonds) can be important factors in terms of their influence on the investor reactions to green bond announcements.

*H4*: Reactions to issuers that are listed on both markets will depend on their dominant institutional dynamics (i.e., strategic framing of the announcement and source credibility of the issuer) in both markets.

When an announcement is framed with dominant institutional logic, it leads to positive stock market reaction (Rhee and Fiss, 2014). Institutional logic can be described as being in line with the strategy of a firm to transition to the green economy, such as through the issuance of a green bond or other green firm-level initiatives (Saravade and Weber, 2020). Rhee and Fiss (2014) found that effectiveness of framing (or how aligned with institutional logic) and source credibility (the communicator of the announcement) depend on the institutional attributes such as announcer visibility, prior performance, and practice prevalence (Rhee and Fiss, 2014).

It seems that having greater public focus on the announcement, with an emphasis on the prior visibility and financial performance of the issuer, can be beneficial in terms of the stock market reaction. This is supported by Wang et al. (2020), who show how stock market investors in China already react positively to green bond issuance news. Based on this literature, we hypothesize that firms having better strategic framing of their announcements, i.e., those in line with their green initiatives or business mandates, and those having better source credibility, i.e., green industry firms, will have a better stock price reaction after the announcement of their green bonds.

So far there has been a lack of contextual analysis for market reactions to green bonds, which means firms or issuers may not be sure why reactions occur and what characteristics may be important at the firm and bond level. Our paper looks to address this literature gap by linking the institutional dynamics of the green bond market to its stock market reactions. This is important because it has been assumed that only “hard laws” like regulations can work in China, whereas Hong Kong’s open market system needs more a “soft power” approach in influencing green bond market growth (Huang and Yue, 2020) and its overall legitimacy (Saravade and Weber, 2020). Our paper examines this assumption by comparing the two stock market reactions after green bond announcements. Hence, it fills the gap through the identification of the institutional dynamics that might influence these reactions.

## Sample and Methods

### Sample Selection

We selected the samples according to the following steps: First, we identified all green bond issuances from 2016 to 2019 in China. Second, we picked the green bonds that were issued for the first time by a single issuer in the same year. For example, some issuers listed green bonds more than once in the same year. Hence, in order to capture the unique reaction after a first announcement, we only counted the first announcement per issuer in that year. This left us with a sample of 350 announcements. Third, we removed any issuances that were by non-listed firms, which left us with a smaller sample of 93 unique green bond announcements by listed firms including banks. Fourth, some bond issuers were listed later than the announcement date of green bond issuance, which means they are lack of stock dealing data. After we delete these issuers, there are 82 samples left. Table 1 provides a summary of our sample. All the data were collected from Wind Database (in Mainland China) and cross-checked with available data from China Bond Information Network and China Finance Information Network.

**Table 1:** Sample structure and related steps.

Step1	All green bonds during 2016 to 2019	764
Step2	Green bonds issued by different firms the first time in the same year	350
Step3	Green bonds issued by listed firms	93
Step4	Green bonds issued by listed firms without stock dealing data missing	82

In terms of evaluating the data, we categorized banks and non-bank companies separately due to differences in capital direction. Since the main business of a bank is to lend or invest into other businesses or projects, whereas non-bank issuers mainly use any capital to provide new or updated products or services. Given this distinction, it was necessary to distinguish them as their green bond issuances might have led to capital being directed in different ways, and this would be reflected by investor preferences. Secondly, company ownership was an important factor based on its ability to affect environmental performance and investor reaction in China (Weber, 2017; Yin, 2017). Hence, ownership majority was examined, and issuers were split into government-owned and private-owned firms. Thirdly, given the literature on the influence of bond-level characteristics, we chose to examine different types of bonds, namely general corporate bonds, financial bonds, and non-general bonds. Fourthly, certain industry-level characteristics like green industries versus non-green industries could also affect the demand and institutional standing for companies that issue green bonds. We referred to the standards of the Green Industry Guidance Catalog to identify the green industries.

### Methods

We evaluated the stock investors’ reaction toward the issuance of green bond by using the event study method. The event study method can be used to assess whether or not a green bond issuance is an unexpected event or presents novel information for the stock investors. Previous research supports the use of event study methods to understand how investors react to a particular event (Song and Han, 2017; Ozo and Arun, 2019). To establish the basis of our event study, we set the parameters as follows:

1. Event date: We defined the date when the issuer first announced the green bond issuance as event date (*d* = 0).
2. Event window: We choose *d* = [−3, 7] as the event window.
3. Estimation window: Campbell et al. (1997) point out that for an event window of (−30, +30) or less, the estimated window can be 120 days or longer. Therefore, this paper selected (−155, −6) a total of 150 trading days as the estimation window.
4. The normal rate of return estimation model: Brenner (1979) highlights that the simplest market model is as good as other complex models. Hence, we chose the market model as the predictive model of the normal stock return.
5. Rate of return: The daily return rate of individual stocks is the rate of return after considering factors such as cash dividends, bonus shares, and allotment. For the market index rate of return, we chose the Shanghai Stock Index (000001) for the Shanghai stock market sample, the Shenzhen Stock Exchange Index (399106) for the Shenzhen stock market sample, and the Hang Seng Index (HSI) for the Hong Kong sample. According to the above standards, we calculated the daily abnormal return (AR) and cumulative abnormal return (CAR) of each sample company in the (−3, +7) time window.

## Results

### Descriptive Statistics

Our descriptive statistics consists of 72 individual issuers that issued a total of 82 green bonds between 2016 and 2019. To better understand the samples, we used three categories (as shown in Figure 2). There were 58 non-banks (bank = 0) and 35 banks (bank = 1) in our sample. However, non-bank issuers had the majority and were growing year by year. In terms of ownership, there were 78 state-owned firms (gov = 1) and 15 private firms (gov = 0) in our sample. Based on the types of bonds, we had a total of 43 general corporate bonds (type = 1), 32 financial bonds (type = 2), and 18 non-general bonds (type = 3, including ABS, short-term bonds or notes). In terms of type of industry, we had 25 environmentally friendly firms (ind = 1) and 68 other firms (ind = 0).

### Test of Hypothesis

We tested market reaction caused by the issuance of green bonds based on different years and stock markets. Due to social unrest protests in Hong Kong in 2019, financial markets like the stock market were most likely affected as well. Hence, when looking at the overall effect, we considered whether to exclude 2019 in both cases. However, our test results in Table 2 showed that 2019 was no different and it was ultimately included in our analysis.

**Table 2:** Green bonds CAR calculated in both markets.

	Mainland China Stock Market			Hong Kong Stock Market		
Year	Samples	Coef. for CAR [−3,7]	T-test for CAR [−3,7]	Samples	Coef. for CAR [−3,7]	T-test for CAR [−3,7]
2016	5	0.0103	4.1982^***^	6	0.8714	2.0123^**^
2017	11	0.0106	1.9449^*^	9	1.2608	5.1729^***^
2018	14	−0.0084	−1.1890	10	2.2275	6.0890^***^
2019	22	−0.0040	−2.2862^**^	10	0.3590	0.8466
2016–2018	30	0.0024	1.8905^*^	25	0.6463	3.0022^***^
2016–2019	52	0.0014	1.8425^*^	35	0.5585	2.8254^***^

*It is sorted out according to the data analysis results. Similarly, hereinafter*. *p < 0.1, **p < 0.05, and ***p < 0.01.

#### Test Result for H1

For Mainland China’s stock markets, we got an overall significant positive CAR value across the entire sample period. Among them, the CAR value was positively significant in the first 2 years, but not significant in 2018, and negatively significant in 2019. For Hong Kong stock market, the situation was similar, except for positive significance in 2018 and no significance in 2019. However, if we take the overall period from 2016 to 2019 for both Hong Kong and Mainland China, the overall results were significantly positive. The results here show that H1 is confirmed and that there was an overall positive reaction toward green bond announcements in both stock markets.

In order to understand whether this reaction was unique to green bonds, we compared it with non-green bonds issued by non-banks by using the method of propensity score matching (PSM). The matching variables include the industry of the firms, the issuing date of the bonds, the amount of the bonds, and the maturity of the bonds. As mentioned in Table 3, most of the results were not significant, but the T-value of the CAR difference between treat group and control group was mostly positive, whether it was a 1:3 or 1:1 pairing method. The results provided further evidence for H1, meaning that green bond announcements had a more positive reaction as compared to non-green bonds.

**Table 3:** Non-green bonds in Mainland China.

1:3 nearest-neighbor PSM method				1:1 nearest-neighbor PSM method			
dif	CAR (mean)		T value (treat-control)	dif	CAR (mean)		T value (treat-control)
	treat	control			treat	control	
−5	0.0286	−0.3640	1.3956	−5	0.0386	−0.6358	1.9754^**^
−4	0.3867	−0.6725	1.7330^*^	−4	0.3481	−0.3253	1.8755^*^
−3	−0.2086	−0.7548	0.7410	−3	−0.5953	−0.6800	0.1887
−2	−0.0142	−0.6840	0.7354	−2	0.1944	−0.1372	0.8999
−1	−0.3363	−1.0407	0.6740	−1	−0.3221	0.0122	−0.7289
0	−0.2909	−1.3452	0.9184	0	0.0454	−0.1652	0.4903
1	−0.0604	−1.3381	1.0751	1	0.2305	0.1918	0.1040
2	0.2592	−0.1305	1.2663	2	0.3196	−0.0952	1.6246
3	0.1061	−1.5307	1.2191	3	−0.1531	−0.3253	0.4824
4	0.2855	−1.3036	1.0594	4	0.1795	−0.1603	0.6269
5	0.2034	−1.3665	1.0344	5	−0.0821	−0.0747	−0.0199
CAR	CAR (mean)		T value (treat-control)	CAR	CAR (mean)		T value (treat-control)
	treat	control			treat	control	
CAR1	0.0454	−0.3045	0.7779	CAR1	0.0454	−0.1652	0.4903
CAR3	0.0502	−0.6566	0.9279	CAR3	−0.0299	0.1582	−0.2534
CAR5	0.5474	−0.5503	1.2636	CAR5	0.4835	−0.0938	0.6685
CAR7	−0.2800	−0.6791	0.3510	CAR7	−0.3908	−1.1950	0.7739
CAR9	0.2671	−0.6388	0.6240	CAR9	0.0933	−1.5989	1.1710
CAR11	0.2035	−1.3665	1.0344	CAR11	0.2034	−2.3949	1.6244

#### Test Result for H2

**Figure 3:**
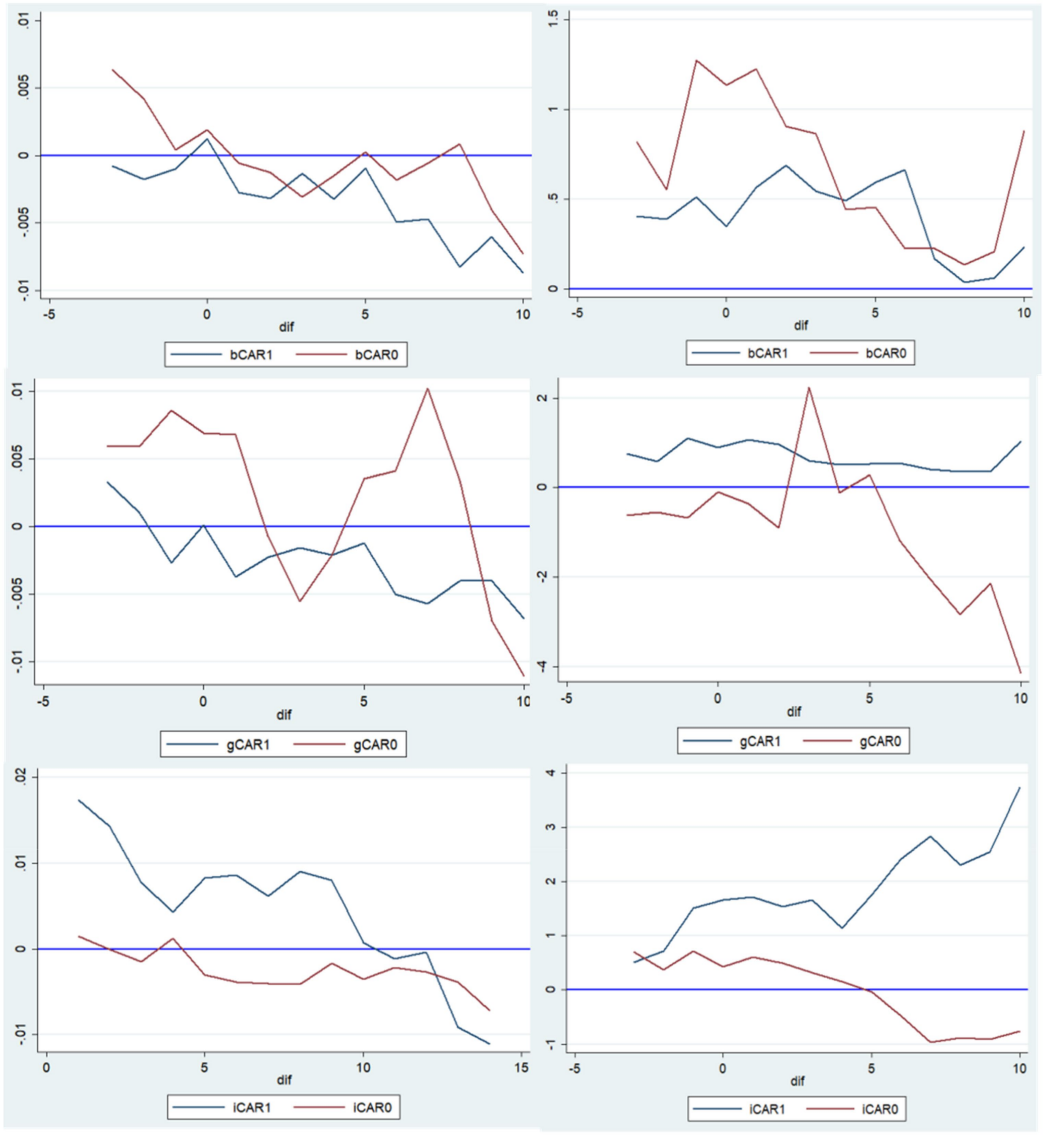
Comparison of CAR in two stock markets (Left: Mainland China, Right: Hong Kong).

Our second hypothesis stated that reactions in the two markets could be influenced due to issuer characteristics (like banks vs. non-banks, ownership type and industry type). As highlighted by Figure 3, we mapped the CAR value comparisons based on the firm characteristics of bond issuers. On the horizontal axis are the various dates, with zero representing the event date or the actual date that the listed issuer announced their green bond. On the vertical axis, we have the value of CAR; bCAR1, bCAR0, gCAR1, gCAR0, iCAR1 and iCAR0 are the mean values within the estimation window of [−3,7], where the issuer is a bank, a non-bank firm, a state-owned firm, a private firm, an environment-friendly firm, and a common firm, respectively.

For Mainland China market, non-bank issuers got a more positive and significant CAR value than bank issuers, private firm issuers got a positive but not significant CAR value than state-owned issuers, and environmentally friendly issuers got a more positive and significant CAR value than other issuers. The t-value results of the CAR difference test between the three groups were 2.1143, 0.9611 and 1.8705, indicating that for issuer type and industry type the values were more positive and significant.

For Hong Kong market, non-bank issuers got a positive significant CAR value than bank issuers, state-owned issuers got a more positive but not significant CAR value than private issuers, and environmentally friendly issuers got a more positive significant CAR value than other issuers. The t-value results of the CAR difference test between the three groups were 2.0016, 1.1039, and 1.9804, indicating that for issuer type and industry type the values were more positive and significant.

Hence, our hypothesis here was partly confirmed as there were more common variables that held than anticipated. For instance, both markets had a more positive reaction to non-banks as well as environmentally friendly firms. However, the ownership aspects were not significant and differed in the two contexts—with Mainland China having a more positive reaction to private firms and Hong Kong having a more positive reaction to state-owned issuers.

#### Test Result for H3

**Figure 4:**
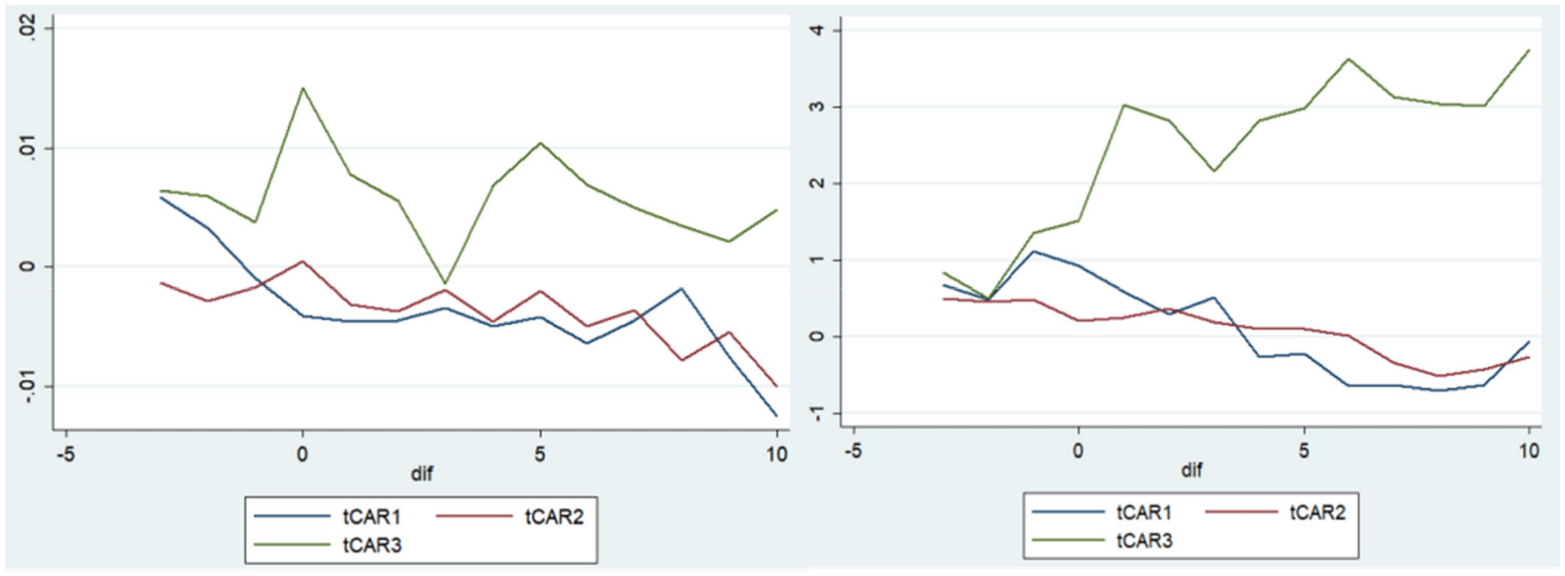
CAR comparison based on type of bonds (Left: Mainland China, Right: Hong Kong).

Our third hypothesis stated that the reactions in the two markets differed based on bond characteristics (like general corporate, financial, and non-general bonds). Figure 4 shows the CAR value comparison result based on the type of bonds (Left—Mainland China, Right—Hong Kong). The tCAR1, tCAR2, and tCAR3, are the mean value of all the CAR within the estimation window of [−3,7] where the bond is a general corporate bond, a financial bond, and a non-general bond (such as ABS and medium-term notes), respectively.

We can see that the CAR value of non-general bonds was more positive in both markets. In order to test between the groups, we tested the difference of CAR value between type = 1 and type = 2&3, with the t-value was 3.0506. However, other group test results for differences between groups were not found to be significant. This partly confirmed our H3 that reaction was different based on bond characteristics, but a more common positive and significant reaction existed for non-general bonds than other types of bonds.

#### Test Result for H4

Our fourth hypothesis stated that positive reactions for firms listed in both markets would depend on their dominant institutional dynamics (i.e., strategic framing of the announcement and source credibility of the issuer). For this sample, we had a total of 13 green bond announcements across the time period chosen, coming from 10 different firms (7 bank issuers and 3 non-bank issuers) listed in both Mainland China and Hong Kong stock markets.

Some issuer-level data were missing because the date of the firm listed on the stock market was later than the announcement data of their green bond. The CAR values were tested for each firm, and Table 4 shows the result, where the companies coded as JFKJ, BYD, and DSHJ were non-bank issuers and the rest were banks. We found that the reaction was similar for each firm in the two markets; although some were not significant, the direction was the same. Most banks got a positive significant result; however, the situation for non-banks was the opposite. Interestingly, the only positive reaction for a non-bank was for a wind turbine manufacturing company. This confirmed our H4 that hypothesized differences in the reactions for both markets, but certain issuers having better framing of announcement and source credibility in terms of green bond (like a wind turbine manufacturing company) would get a more positive reaction in both markets. The explanation for this will be discussed in the next section.

**Table 4:** Chinese firms listed in both stock markets.

Company code	Mainland China Stock Market		Hong Kong Stock Market	
	Coef. for CAR [−3,7]	T-test for CAR [−3,7]	Coef. for CAR [−3,7]	T-test for CAR [−3,7]
JFKJ16	0.0130	4.0653^***^	3.6435	3.3617^***^
BYD18	−0.0252	−1.4542	−1.5682	−1.9007^*^
BYD19	−0.0079	−1.2252	−1.3908	−1.1672
DSHJ19	−0.0408	1.6011	2.5411	1.5071
QDYH16			0.9311	2.1603^**^
JTYH16	0.0094	8.8075^***^	1.3332	3.1817^***^
JTYH17	−0.0065	−1.7574^*^	−2.0623	−5.0203^***^
ZZYH17			0.1215	0.2823
NYYH17	−0.0125	−1.7897^*^	−0.0925	−0.5982
ZSYH19	0.0103	1.0144	2.7553	6.8984^***^
ZHSYH19			0.0907	0.3212
JSYH19	0.0191	8.0981^***^	2.4452	5.3368^***^
ZZYH19			4.1249	9.2937^***^

*p < 0.1, **p < 0.05, and ***p < 0.01.

### Test for Robustness

#### Changing the Estimation Window

The chosen model to test the estimation window was the market model, due to its suitability for the Chinese securities market (Brenner, 1979; Chen and Chen, 2002). Based on previous studies, we choose this model as the predictive model of the normal stock return. When we estimate normal returns, using different estimation windows could have different results (Campbell et al., 1997). Hence, upon changing the estimation window, if the final result did not change, it would indicate that our test results were robust. As shown in Table 5, we re-tested H1 by narrowing the estimation window from (−155, −6) to (−95, −5) and found they were robust enough due to the similar results.

**Table 5:** Robustness test results based on changing the estimation window.

Year	Mainland China Stock Market			Hong Kong Stock Market		
	Samples	Coef. for CAR [−3,7]	T-test for CAR [−3,7]	Samples	Coef. for CAR [−3,7]	T-test for CAR [−3,7]
2016	5	0.0017	2.4798^***^	6	1.0077	2.2689^**^
2017	11	0.0064	2.7706^***^	9	1.0233	3.7220^***^
2018	14	−0.0105	−1.1653	10	3.6255	3.6725^***^
2019	22	−0.0075	−3.6776^***^	10	−1.0122	−1.1672
2016–2018	30	0.0024	0.5891	25	1.1391	2.9020^***^
2016–2019	52	−0.0023	−0.9515	35	0.6065	1.9701^*^

*p < 0.1, **p < 0.05, and ***p < 0.01.

**Table 6:** Robustness test results based on replacing stock price with trading scale.

Year	Mainland China Stock Market			Hong Kong Stock Market		
	Samples	Coef. for CATR [−3,7]	T-test for CATR [−3,7]	Samples	Coef. for CATR [−3,7]	T-test for CATR [−3,7]
2016	5	0.2011	12.0998^***^	6	0.7065	8.2890^***^
2017	11	0.1906	7.6008^***^	9	0.9022	4.2701^***^
2018	14	0.1150	1.0536	10	1.1650	2.9724^***^
2019	22	−0.0975	−0.9064	10	−1.0013	−1.5317
2016–2018	30	0.1024	1.9581^*^	25	1.0319	3.0901^***^
2016–2019	52	0.0305	1.0955	35	0.8803	1.9004^*^

*p < 0.1 and ***p < 0.01.

#### Replacing Stock Price With Trading Scale

Similarly, a second test for robustness was to use a stock trading scale; which reflects the willingness of stockholders to either sell stocks or for potential investors to buy the stocks (Huang et al., 2010). This test is based on the assumption that when investors have positive expectations of a certain firm, they are very likely to increase their stock holdings. Hence, this test could also represent the attitude of stock investors to a particular firm or stock. In order to test whether stock investors had positive reactions to green bond announcements, we replaced CAR with ATR (that calculates abnormal rate of trading scale) and re-tested H1. The results were similar to those in Table 6, which meant that they were confirmed to be robust.

## Discussion and Conclusion

### Discussion

Taking the stock market reaction of Mainland China and Hong Kong to corporate green bond announcements, we used the event study method to test whether the response was an overall positive one, and if so, what was the impact of issuer- or bond-level characteristics on this reaction. By comparing two different sociopolitical regions, we also wanted to test whether there was a role of institutional influences on the stock market’s reaction to green bonds. The empirical test results helped us analyze how these stock markets reacted to green bonds. Firstly, green bonds announcements from firms led to an overall positive reaction in both stock markets. This was important because it reinforces the notion that green bonds were seen as having not just the signaling ability around the future of green finance for its investors, but also serves to convey positive information about an issuer’s financial and environmental performance in the market (Monasterolo and Raberto, 2018; Broadstock and Cheng, 2019; Chiesa and Barua, 2019; Flammer, 2021).

Given that stock investors usually vote with their feet and are swayed by information sentiment (Akhtar et al., 2012), there is reason to believe that they might have positive future expectations for companies issuing green bonds. Such confidence may arise from the policy development prospects for a green economy (Weber, 2017; Monasterolo and Raberto, 2018), or may come from investors’ preference for environmental protection and CSR (Cordeiro and Tewari, 2015). Furthermore, institutional policy signals from major government announcements in recent years show that the Chinese government is vigorously promoting and encouraging green technology innovation and supporting green enterprises, giving investors’ further confidence in the development of shared green industries in the region. However, for the investors based in the Hong Kong stock market, it might not necessarily be driven by the institutional policies of the government as seen by the fairly new guidance and policies of Hong Kong regulators for this market. However, the overall positive response to the issuance of green bonds in Hong Kong may come from their inherent investor preferences for green finance, which is grounded in the growth of real estate green assets as well as Hong Kong’s position of being a major global finance center that houses several international companies and financial institutions.

Secondly, the two stock markets also responded to green bond issuance based on firm- or issuer-level characteristics like type of issuer and industry type. For both Mainland China and Hong Kong markets, non-banks and environmentally friendly firms got a more positive and significant reaction. However, for ownership characteristics, Mainland China’s stock markets reacted more positively toward private issuers, whereas Hong Kong’s stock market reacted positively toward state-owned issuers. Interestingly ownership was not seen as being significant in the results, whereas characteristics like issuer type and industry type played a much more important role for investors.

In terms of explaining the differences in issuer-type characteristics, the perception for non-banks issuers could be that they might not have enough sources of financing and may need to be more innovative through the use of green bonds for raising capital. Furthermore, when non-banks issue a green bond, it is usually related to green projects that were ongoing or upcoming and could be measured in terms of a direct impact, whereas when banks issue a green bond, it could be redirecting the money toward credit activities that may or may not be directly measured or linked to a specific project. Based on a green bond’s emphasis of providing measurable financial benefits and green impacts, announcement of a green bond could influence how stock investors approach the potential of a listed issuer to use the capital toward innovative projects that ultimately help improve the stock value and reputation of the company.

In terms of explaining the positive reaction for private companies from Mainland China’s stock market, it could be attributed to the fact that they are seen as being more innovative than state-owned issuers, who are more likely to follow prescriptive policies (not seen as organic in a market setting, but more as fulfilling political criteria) as set out by the government. Lastly, in terms of preference for environmentally friendly firms, this reaction is in line with literature which indicates that environmental protection companies intuitively showcase greater corporate social responsibility and subsequently show a stock price benefit (Cordeiro and Tewari, 2015). With green bonds having a significant public relations component to it, any corporate signaling information (like green bond announcements) to investors is shown to highlight firm quality and reputation, especially in times of significant change (Kurov, 2010; OuYang et al., 2017), as seen in the current societal and governmental efforts toward the low-carbon transition. Hence, firm-level aspects like issuer type and industry type are seen to matter across both markets. The implications here are that listed issuers having a portfolio mandate of raising green bond capital for specific projects, which are also aligned with popular green sectors and show use of market best practices like third-party reviews, could benefit from the stock market as well. Such outcomes can ultimately influence the internal policies and strategies for listed companies that are looking to be financially successful in the low-carbon economy.

Thirdly, non-general bonds also got a more positive reaction than general or financial bonds. We believe that this was due to non-general bonds having a unique flexibility component aspect to it, given its shorter terms. For instance, stock market investors might feel that they are better able to observe and track the financial performance of such bonds rather than those having long-term maturity dates. With such financial innovations being encouraged by Chinese financial regulators over the years, having flexible financial instruments like non-general bonds can come with certain advantages compared to traditional financial instruments that might not fit the short-term mindset of a stock investor. These bonds not only have short maturity dates and require less financing commitment from their investor; they are also more attractive to investors having smaller assets and short capital recovery cycles (Boubaker et al., 2019). The disadvantages for non-general bonds are that the regulatory policies are less mature as compared to the traditional bond types. However, financial regulatory authorities are now issuing more clear guidelines to encourage their issuance, as seen in the “Rules for the Directional Issuance and Registration of Debt Financing Instruments of Non-financial Enterprises” (China Association of Interbank Market Dealers, 2017). This allows more investors to remain confident about a regulatory oversight being present in the market.

Fourthly, reactions for firms listed in both markets were similar—although some were not significant, the direction was the same for most. This showed that investors in the two markets had the same attitude toward green bonds. Given that our sample consisted of companies listed on both the stock markets at the same time, it reduced the influence of institutional pressures like having different sociopolitical contexts. Therefore, the empirical results suggest that even without the government’s implicit coercive policies (such as that of Mainland China), both investors might have an environmental preference and prefer financial announcements that are in line with global voluntary disclosure practices (as seen in the case of Hong Kong market). Another finding in line with literature is that both stock markets were now merging toward integration in terms of market reactions, and this further legitimized the green bond market becoming more mainstream in the traditional financial sector (Saravade and Weber, 2020). In addition, our study also showed that Mainland China’s financial market reactions were also moving closer to the international trends related to sustainable finance.

In terms of the reactions to issuers having a more dominant standing based on their strategic announcement framing and source credibility, we found that this aspect mattered to stock investors. This was seen in the case of the only non-bank issuer, which was a wind turbine manufacturer and had the only positive reaction for a non-bank issuer in this sample. Based on their strategic framing to issue a green bond in the renewable energy space, as well as having better source credibility due to being from a green industry, it led to the optimal conditions for stock investors to react in a positive way. This was in comparison with the opposite reaction investors who had for other non-bank issuers, which did not have such institutionally aligned dynamics in place. Hence, the role of institutional aspects related to issuer and bond characteristics was seen as being important to influence stock investors perception of a listed firm.

### Policy Implications

Policy implications from our results point to better alignment and coordination of policies or internal strategies to fit within the dominant institutional narrative surrounding climate change and green finance in this region. Issuing a green bond allows listed issuers to get a positive reaction from stock market investors, but certain issuers having business models that directly relate to a green industry are able to gain reputational and financial benefits across both markets. This has two further policy implications: environmental preferences are becoming important among stock market investors, and due to their preferential response to certain bond- and issuer-level characteristics (issuer and industry type), having better policy direction from government and regulators (based on these characteristics) as to where the future financial opportunities lie in the low-carbon economy. This can help support greater financial flows into regional green bond markets and help them evolve in a confident manner that is appealing to investors in both long- and short-term financial markets.

### Conclusion

In financial literature, stock market reaction has been an important indicator to understand investor sentiment and behavior biases when it comes to firm- or event-level information. In our study, we find that green bonds are a unique way to examine how stock market investors can react to information that is more than just about the firm’s financial performance. We find that within a previously unexplored institutional comparison of Mainland China’s top-down policy versus Hong Kong’s free market approach, sociopolitical differences do not seem to hinder the popularity of the green bond market across the financial sector. Our results reinforce previous literature that shows green bonds have a positive influence on stock market price for the listed issuer. Furthermore, we undertake two additional tests for robustness to confirm our results in the case of Mainland China and Hong Kong.

Our specific contribution comes in the form of examining whether specific issuer-level characteristics (issuer and industry types) or specific bond-level characteristics (bond types) will have a positive impact in terms of being seen as attractive information for a stock investor. Our results show that such issuer- and bond-level characteristics can elicit a positive reaction across both institutional settings, meaning that investors in both markets agree on the value-add of a green bond. However, certain institutional dynamics like strategic framing of green bond announcement and source credibility of an issuer will have a more positive impact when it comes to the stock price increase and for investor reaction in the market.

Based on our current results, we find that it is important for the broader financial community to recognize that green bonds are here to stay and provide a positive stock market benefit to those issuing them. Although our contributions are novel in using green bond announcement to examine investor sentiment, our study did face certain limitations. First, there were challenges with data availability due to Mainland China’s market being less mature, as well as limited access to rich qualitative interview data regarding what was driving the institutional perceptions of investors. We suggest future research to examine these aspects within more matured market contexts or undertake a mixed methods approach to fill the gaps. Second, we refer to investor sentiment by abnormal volatility in short-term stock prices. However, debate remains as to whether volatility in stock prices is indicative of investor sentiment. We look forward to finding a better and uncontroversial way to measure investor sentiment in the future.

## Data Availability Statement

Publicly available datasets were analyzed in this study. This data can be found at: https://www.wind.com.cn/.

## Author Contributions

XC conceived of the early stage idea. XC and OW developed the theory and performed the computations. XC and VS verified the analytical methods. All authors discussed the results and contributed to the final manuscript.

## Funding

This work was supported by Science Foundation for Young Teachers of Wuyi University.

## Conflict of Interest

The authors declare that the research was conducted in the absence of any commercial or financial relationships that could be construed as a potential conflict of interest.

## Publisher’s Note

All claims expressed in this article are solely those of the authors and do not necessarily represent those of their affiliated organizations, or those of the publisher, the editors and the reviewers. Any product that may be evaluated in this article, or claim that may be made by its manufacturer, is not guaranteed or endorsed by the publisher.
